# Belgian hand hygiene campaigns in ICU, 2005–2015

**DOI:** 10.1186/s13690-016-0159-3

**Published:** 2016-11-07

**Authors:** Sylvanus Fonguh, Annie Uwineza, Boudewijn Catry, Anne Simon

**Affiliations:** 1 Scientific Institute of Public Health, Operational Direction Public Health and Surveillance, Healthcare Associated Infections and Antimicrobial Resistance Unit, Brussels, Belgium; 2 Cliniques Universitaires Saint-Luc UCL, Laboratory of Microbiology, Brussels, Belgium; 3 Pôle de Microbiologie, Institut de Recherche Expérimentale et Clinique, UC Louvain, Brussels, Belgium

**Keywords:** Hand hygiene compliance, Healthcare-associated infections, Intensive care units

## Abstract

**Background:**

Healthcare-associated infections (HCAI) are still a major problem especially in most intensive care units (ICU). Incompliance by clinical staff with hand hygiene (HH) increases rates of preventable infections. We report the outcome of the Belgian national hand hygiene campaign from 2005 to 2015 with focus on intensive care units.

**Methods:**

Using the World Health organisation (WHO) standardised observation roster, trained infection control teams measured adherence to HH guidelines by direct observation. HH opportunities were counted and the actual episodes of HH were scored as no HH, HH with water and soap, or HH with alcohol-based hand rub. Measurements were repeatedly done before and after a one month awareness campaign every second year. Compliance was stratified by indication and by type of healthcare worker, and computed as a percentage of the number of HH episodes with water and soap or with alcohol-based hand rub, divided by the number of opportunities.

**Results:**

A total of 108,050 hand hygiene opportunities were observed in ICU during this period. HH compliance increased significantly from 49.6 % before campaign in 2005 to 72.0 % before campaign in 2015. Over the same time frame, post campaign compliance increased from 67.0 to 80.2 %. The number of opportunities observed substantially increased when automated feedback was installed.

**Conclusions:**

In Belgian intensive care units, hand hygiene compliance is getting improved overtime, though consecutive campaigns with immediate feedback are required to achieve and sustain a high compliance rate.

## Background

For several years, there is evidence that hand hygiene compliance is essential for the prevention of healthcare-associated infections HCAI [1, 2]. Patients admitted in intensive care unit ICU are severely affected and are often immune-compromised. They therefore require heavy care, amongst which include invasive treatments, mechanical ventilations, vascular catheters, thus requiring more complex follow ups than patients of other services [3].

In Europe, the highest rate of HCAI is found in intensive care units [4–7]. In Belgium, at least 6 to 7 % of hospitalised patients per year contract HCAI [5, 8]. According to the point prevalence survey by the European center for disease prevention and control in 2011, the prevalence of HCAI in Belgian ICU was 20.3 % [4]. The national results of the monitoring of HCAI in intensive care units from 2001 to 2014 showed that patients in intensive care are increasingly an aging population, with a high disease severity as measured by their Simplified Acute Physiology Score (SAPS II), and a majority is treated with antimicrobial agents. The 2014 results show that, the median SAPS II score for patients admitted in ICU is greater than 20 thus implying an increase in hospital lengths of stay, infections rates, hospital cost and mortality. [9] That said it becomes essential to emphasise the promotion of good hand hygiene practices in healthcare settings especially in ICU’s.

Belgium is one of the pioneer countries that implemented the WHO “Clean care is safe care” initiative in 2005 [10] by launching the countrywide hand hygiene campaign entitled “You are in good hands” [8]. These campaigns are supported by the Federal Platform for Infection Prevention & Control (FPIPC) and the Belgian Antibiotic Policy Coordination Committee (BAPCOC), with funding from the Belgian federal government, and organised by a multidisciplinary working group.

We here present the result of the national hand hygiene campaign in Belgium from 2005 to 2015, focusing on intensive care units.

### Methodology

Campaigns to promote hand hygiene in Belgian hospitals have been organised since 2005. Hospital participation in these campaigns was voluntary. For every participating acute care hospital, at least observation data in ICU needed to be provided. The Belgian campaign is multimodal, using varied materials (posters, reminder in wards, training sessions for healthcare workers (HCW), patient education and video clips) [8]. Each campaign carried a specific message inspired by the results of the previous campaign, and geared towards improving compliance to hand hygiene (Table 1).

**Table 1:** Evolution of Belgian hand hygiene campaign messages in hospitals

Campaign	Year	Message
First	2005	Hand Hygiene, just di it…and with alcohol rubs
Second	2006–2007	Hand Hygiene; do it correctly
Third	2008–2009	Hand Hygiene, without jewels and with appropriate use of gloves
Fourth	2010–2011	Doctor, don’t forget, it works and you are a model
Fifth	2012–2013	Hand Hygiene, do it certainly before any contact with the patient
Sixth	2014–2015	Hand Hygiene, together with patient

The infection control (IC) teams of participating hospitals were responsible for the implementation of the campaign at their institution. Measurements for each campaign were done *before* and *after* a one month awareness period. The observation periods before and after campaigns were always one month for all campaigns except for the sixth campaign where the observation periods were 2 months 3 weeks before and 2 months after campaigns due to the Ebola crisis.

Using a standardised observation roster, trained infection control teams measured adherence to HH guidelines by direct observation. Hand hygiene compliance data was entered in NSIHwin (MS Access application) until the fourth campaign. From the fifth campaign, data was entered directly using mobile devices or observation roosters developed according to WHO guidelines [11] into an online password protected tool (NSIHweb II) with real-time feedback to the hospitals [12]. Data analysed here are only for those ICU wards with more than 150 observed opportunities per observation period.

HH opportunities were counted and the actual episodes of HH were scored as no HH, HH with water and soap, or HH with alcohol-based hand rub. If both disinfection procedures were applied, it was scored as HH with alcohol-based hand rub. Compliance was stratified by the WHO five moments for hand hygiene in health care/indication (before patient contact, after patient contact, before an aseptic task, after body fluid exposure risk, after contact with patient surroundings) [10] and by type of HCW (nurses, nursing assistants, physicians, physiotherapists, other) and was computed as a percentage of the number of HH episodes with water and soap or with alcohol-based hand rub divided by the number of opportunities. Results were reported by participating hospital as a weighted mean, therefore adjusting for varying number of observed opportunities between hospitals. All data were processed and analysed using SAS 9 software. Comparison of compliance rates between periods and groups were performed using the Wilcoxon signed rank test with a two sided p value <0.05 considered to be statistically significant.

## Results

**Fig. 1:**
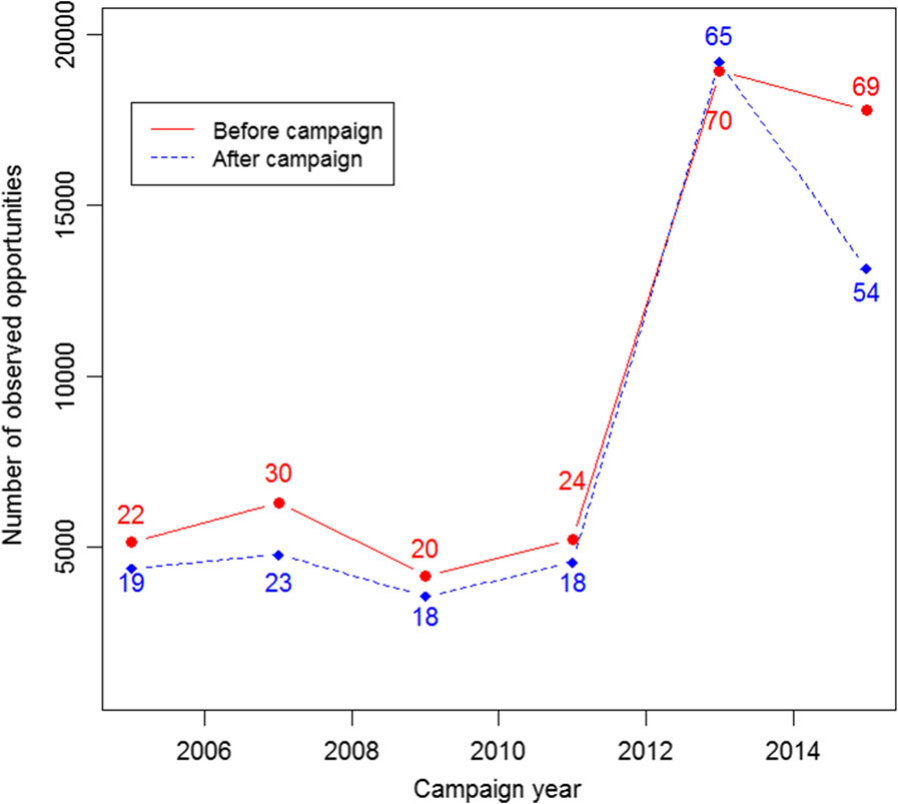
Evolution of number of opportunities over time as recorded by intensive care units (ICU) from hospitals participating in Belgian hand hygiene campaigns. *Legend:* *Dots represent the number of opportunities, while numbers inside the plot represents number of hospitals with more than 150 opportunities observed per ward

Over consecutive years, the number of hospital sites providing ICU data with more than 150 observed opportunities increased from 22 (ca 17 %) to 69 (51 %) before campaign and from 19 (ca 15 %) to 54 (40 %) *after* campaign (Fig. 1) Precise data on number of hospital sites with intensive care units are only available from 2011. The number of observed opportunities also increased from 5149 to 18775 before campaign and from 4358 to 13121 after campaign (Table 1). The highest number of opportunities was recorded in 2013, making a total of 108,050 observed opportunities over the 6 campaigns (Fig. 1).

**Fig. 2:**
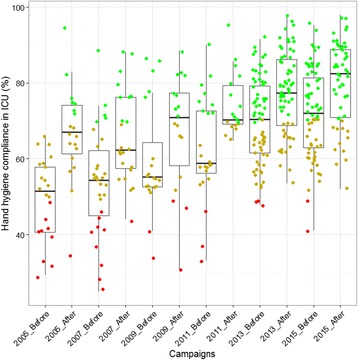
Evolution of Compliance in Belgian intensive care units (ICU) from 2005 to 2015

Overall compliance to hand hygiene (national weighted mean of all hospital sites with more than 150 opportunities per ICU ward combined) increased significantly (*P < 0.05*) for the 2005, 2006, 2011, 2013 and 2015 campaigns (Fig. 2 and Table 2). Though there was an increase from 59.6 to 67.3 % during the 2009 campaign, the increase was not statistically significant. National compliance also increased overtime for both before and after campaigns thus affirming the need for a continuous reminder and the importance of repeating campaigns (Table 2).

**Table 2:** Rates of hand hygiene compliance (%) from 2005 to 2015 in Belgian intensive care units (ICU) participating in nationwide hand hygiene campaigns

	Campaign 2005		Campaign 2007		Campaign 2009		Campaign 2011		Campaign 2013		Campaign 2015	
	Before	After	Before	After	Before	After	Before	After	Before	After	Before	After
Opportunities (N)												
N observed opportunities (ICU)	5149	4358	6310	4776	4167	3548	5221	4553	18898	19174	18775	13121
Compliance rates												
Overall national compliance (%)	49.6	68.6	53.2	69.5	58.0	69.4	62.3	72.9	64.1	75.8	69.1	77.7
Overall ICU compliance (%)	49.6	67.0	53.7	65.9	59.6^a^	67.3^a^	62.7	74.2	69.8	77.5	72.0	80.2
By type of healthcare worker												
Nurse	53.8	69.2	59.5	68.8	63.1^a^	73.3^a^	65.8	76.7	73.0	80.1	75.2	81.8
Nursing assistant	53.7^a^	55.3^a^	60.0^a^	57.6^a^	44.3^a^	76.8	66.2	75.1	67.1^a^	77.7^a^	80.0^a^	75.8^a^
Physician	40.4	57.7	38.5	56.3	53.1^a^	53.5^a^	54.5	56.6	59.0	65.9	61.2	70.6
Physiotherapist	51.8^a^	70.2^a^	57.1^a^	68.0^a^	62.7^a^	71.6^a^	63.6	78.1	73.0	77.9	72.4	79.3
Other	25.8	70.9	33.8^a^	45.6^a^	55.1^a^	47.8^a^	45.2^a^	52.8^a^	53.5	68.6	63.5	74.1
By hand hygiene indication												
Before patient contact	39.3	54.0	44.8^a^	60.7^a^	50.5^a^	59.6^a^	52.2	68.6	62.7	71.5	64.1	75.2
After patient contact	64.3	80.5	68.2^a^	77.6^a^	71.3^a^	78.1^a^	76.3	82.1	81.8	86.0	82.6	87.8
Before aseptic procedure	34.0	48.7	36.9^a^	47.1^a^	43.3^a^	53.3^a^	45.7	64.4	56.9	65.8	59.1	70.7
After body fluid exposure	64.4	85.1	65.0^a^	70.0^a^	67.6^a^	81.1^a^	72.6	84.4	77.1	85.1	77.6	82.5
After contact with patient environment	45.3	61.9	50.0^a^	60.0^a^	55.7	65.1	64.8	65.4	65.6	77.6	69.9	76.8

^a^No significant difference

Compliance rates also increased over time for all types of HCWs with the best performance noted for nurses (81.8 %). Physician compliance did improve significantly from 2011 and was stable around 60 % though significantly (*p < 0.001*) lower than among nurses during all campaigns. There was no significant difference among all HCW’s during the 2009 campaign (Table 2).

Same as for HCW’s, there was an increase in compliance for all WHO indications for hand hygiene over the years. This increase was always championed by the *after patient contact* and *after body fluid exposure* indications compared to *before patient contact* and *before aseptic fluid exposure* indications. A statistical significant increase was observed during the 2005, 2011, 2013, and 2015 campaigns (Table 2). Comparing compliance between before indications and after indications within campaign periods showed that compliance after contact was significantly higher than compliance before contact (*p < 0.001*).

## Discussion

Compliance with hand hygiene, in combination with other infection control measures, significantly reduces the rate of HCAI particularly in intensive care units where care is complex and requires close contact with patients [13, 14]. In Belgium, the hospital wide incidence of nosocomial methicillin-resistant Staphylococcus aureus (n-MRSA) has decreased significantly since the start of nationwide hand hygiene campaigns in 2005, from 4 n-MRSA/1000 admissions to 1.2 n-MRSA/1000 admissions in 2014 [15]. Though this decrease cannot be solely attributed to the campaigns, their impact cannot be overlooked. In 2014, the incidence of ICU-acquired pneumonia and bloodstream infection (BSI) ventilation-associated pneumonia (VAP), based upon a cohort of 13 Belgian hospitals averaged at 6.7 pneumonias and 1.9 BSI per 1000 patient days, respectively. For these mandatory and other optional outcome indicators not reported, this is a decrease as compared to previous years [9].

Comparing the effect of all campaigns over time yielded an increase in HH compliance at short and long term, indicating the importance of regularly repeating campaigns over time, as documented by other studies [2, 8].

The drastic increase in the number of observed opportunities in 2013 and the attainment of the 70 % compliance margin could be explained by the introduction of a free web tool for data entry with real-time feedback to hospitals, nonetheless there was a drop in the number of opportunities in 2015. This could be attributed to the Ebola outbreak in Africa that imparted an extra burden on infection control teams in Belgium in view of preparedness.

Hand hygiene compliance rates were significantly higher after patient contact than before patient contact thus implying that HCWs tend to clean their hands only when perceived as dirty, or they try to protect themselves rather than protect the patients as already reported by other studies [8, 16].

Consistently over all six campaigns, there was a significant difference in compliance between nurses and physicians; with nurses always doing better than physicians in both before and after campaigns. This is not different from the results of other European countries [16–18] but also non-European countries such as Australia or Korea [7, 19, 20]. National compliance in Belgium and other European countries have improved since 2005 and stabilize around 70 % [16, 21]. Various studies have been conducted to understand the attitudes of healthcare workers in relation to the observance of hand hygiene, and results show that poorer compliance among physician cannot be related to lack of knowledge of national or international recommendations or the number of hand hygiene opportunities, but most likely related to difficulties in behavioral change amongst physicians [19]. The campaign message for the fourth campaign focused on physicians as role models. Albeit the fact that nurses still performed better, a marked significantly sustained improvement was recorded for physicians since the fourth campaign. The low compliance of physicians is a global issue, for instance in Australia in 2014, after noticing the same problems, physicians were targeted with a campaign message “doctor do you have a moment?”, and physician compliance was above 61 %.

Like other countries, Belgium uses direct observation with real time feedback to allow caregivers to improve on their compliance. Indeed, even if we ignore the Hawthorne effect (also known as observation bias or the tendency for people to change their behavior when they are aware of an observer) [22] which could be considered as a drawback to our study, direct observation provides a clear picture of the typical mistakes and thus gives room for feedback by the observer. A study conducted in 2015 in Germany showed that under observation, HCWs practice hand hygiene 21 times when observed against 8 in the absence of observation [18] while another study conducted one year before in Brazil showed a high compliance rate of 92 % under observation [23].

Our data also showed a steady increase in compliance over time especially from the fifth campaign where the 70 % margin was crossed. This could be attributed to the introduction of online tool with real time feedback at hospital and service levels. A study conducted in 2013 in Belgium in the ICU on the impact of auditing and feedback in preventing central line infections showed that results were best in services where nurses participated once a week at a feedback meeting [24]. This study therefore supports our results showing that feedback in visual form combined with oral presentations are important in improving compliance and fight against healthcare associated infections.

## Conclusions

We conclude that hand hygiene compliance in Belgian intensive care units is improving overtime, though repeated campaigns are required to achieve and sustain a high compliance rate. Furthermore, the use of an online tool with real time feedback, combined with political and financial support from the Belgian Federal Public Service have helped campaigns to be successful with high participating rates and increased observed opportunities and thus increased compliance.

## References

[1] Kathryn B (2012). Impact of a hospital-wide hand hygiene initiative on healthcare-associated infections: results of an interrupted time series. BMJ Qual Saf.

[2] Pittet D, Hugonnet S, Harbarth S (2000). Effectiveness of a hospital wide program to improve compliance with hand hygiene. Infection Control Programme. Lancet.

[3] Cairns S, Reilly J, Booth M (2010). Prevalence of healthcare-associated infection in Scottish intensive care units. J Hosp Infect.

[4] European Centre for Disease Prevention and Control. Point prevalence survey of healthcare associated infections and antimicrobial use in European acute care hospitals. ECDC, Stockholm (2013) http://ecdc.europa.eu/en/publications/Publications/healthcare-associated-infections-antimicrobial-use-PPS.pdf. Accessed 27 Jul 2016.

[5] Gordts B, Vrijens F, Hulstaert F, Devriese S, Van de Sande S (2010). The 2007 Belgian national prevalence survey of hospital-acquired infections. J Hosp Infect.

[6] Salam MF (2013). The effect of hand hygiene compliance on hospital-acquired infections in an ICU setting in a Kuwaiti teaching hospital. J Infect Public Health.

[7] Azim S, McLaws M-L (2014). Doctor, do you have a moment? National Hand Hygiene Initiative compliance in Australian hospitals. MJA.

[8] Costers M, Viseur N, Catry B, Simon A. Four multifaceted countrywide campaigns to promote hand hygiene in Belgian hospitals between 2005 and 2011: impact on compliance to hand hygiene. Euro Surveill. 2012;17(18):pii = 20161. http://www.eurosurveillance.org/ViewArticle.aspx?ArticleId=20161. Accessed 20 Apr 2016. 10.2807/ese.17.18.20161-en 22587957

[9] Scientific Institute of Public Health, Belgium, Surveillance of ICU –Acquired infections National feedback Report, Infections indicators 2014. http://www.nsih.be/download/hi_fbc_nat_2014_12Nov2015_124653.pdf. Accessed 27 Jun 2016.

[10] World Health Organisation. WHO Guidelines on Hand Hygiene in Health Care (Advanced Draft): A Summary. 2005. http://apps.who.int/iris/bitstream/10665/44102/1/9789241597906_eng.pdf. Accessed 19 Apr 2016.

[11] Sax H, Allegranzi B, Chraïti MN, Boyce J, Larson E, Pittet D (2009). The World Health Organisation hand hygiene observation method. Am J Infect Control.

[12] Scientific Institute of Public health – Public health and surveillance. Healthcare Associated Infections (NSIH) Nsihweb 2.0 user manual. Brussels 2014. www.nsih.be. Accessed 20 Jun 2016.

[13] McLaws ML, Pantle AC, Fitzpatrick KR, Hughes CF (2009). More than hand hygiene is needed to affect Methicillin-resistant Staphylococcus aureus clinical indicator rates: clean hands save lives, part IV. Med J Aust.

[14] Kjonegaard R, Fields W, Peddecord KM (2013). Universal rapid screening for Methicillin-resistant Staphylococcus aureus in the intensive care units in a large community hospital. Am J Infect Control.

[15] Scientific Institute of Public Health, Belgium, Surveillances de bactéries résistantes aux antibiotiques dans les hôpitaux belges (MRSA, BLSE+, VRE, CPE+ et A. baumannii-MR et P. aeruginosa-MR): année 2014. http://www.nsih.be/download/MRSA/MRSA_ESBL_CPE_Y2014/RAPPORT_AMR_Y2014_FR.pdf. Accessed 27 Jul 2016.

[16] Wetzeker W (2016). Compliance with hand hygiene: reference data from the national hand hygiene campaign in Germany. J Hosp Infect.

[17] H.Sax et al. My five moments for hand hygiene: a user-centered design approach to understand, train, monitor and report hand hygiene. The Hospital Infection Society; doi:10.1016/j.jhin.2007.06.004. 10.1016/j.jhin.2007.06.004 17719685

[18] Hagel S, Reischke J, Kesselmeier M, Winning J, Gastmeier P, Brunkhorst FM, Scherag A, Pletz MW (2015). Quantifying the Hawthorne effect in hand hygiene compliance through comparing direct observation with automated hand hygiene monitoring. Infect Control Hosp Epidemiol.

[19] Syed Azin et al. An average hand hygiene day for nurses and physicians. The burden is not equal. American Journal of Infection Control 2016; doi:10.1016/j.ajic.2016.02.006 10.1016/j.ajic.2016.02.006 27040570

[20] Seung Soon Lee, Se Jeong Park, Moon Joo Chung, Ju Hee Lee2, Hyun Joo Kang2, Jeong-a Lee1, and Yong Kyun Kim. Improved Hand Hygiene Compliance is Associated with the Change of Perception toward Hand Hygiene among Medical Personnel; http://dx.doi.org/10.3947/ic.2014.46.3.165 10.3947/ic.2014.46.3.165 PMC4189144 25298905

[21] Scientific Institute of Public Health, Belgium, Belgian hand hygiene campaign. National feedback Report, 2015. http://www.nsih.be/surv_hh/download/Résultats%20nationaux%202014-2015.pdf. Accessed 20 Sep 2016.

[22] Srigley JA, Furness CD, Baker GR (2014). Quantification of the Hawthorne effect in hand hygiene compliance monitoring using an electronic monitoring system: a retrospective cohort study. BMJ Qual Saf.

[23] Miguel Almeida O. Filho et Al. Comparison of human and electronic observation for the measurement of compliance with hand hygiene.M.A.O. Filho et al. Am J Infect Control 42 (2014) 1188–92; doi:10.1016/j.ajic.2014.07.031 10.1016/j.ajic.2014.07.031 25234045

[24] Cherifi S (2013). A multicenter quasi-experimental study: impact of a central line infection control program using auditing and performance feedback in five Belgian intensive care units. Antimicrobial Resistance and Infection. Antimicrob Resist Infect Control.

